# Risk Factors for Microvascular Complications of Diabetes in a High-Risk Middle East Population

**DOI:** 10.1155/2018/8964027

**Published:** 2018-07-02

**Authors:** Sohaila Cheema, Patrick Maisonneuve, Mahmoud Zirie, Amin Jayyousi, Hekmat Alrouh, Amit Abraham, Sura Al-Samraye, Ziyad Mahfoud, Ibrahim Mohammed Al-Janahi, Buthaina Ibrahim, Albert B. Lowenfels, Ravinder Mamtani

**Affiliations:** ^1^Institute for Population Health, Weill Cornell Medicine-Qatar, Education City, Qatar Foundation, P.O. Box 24144, Doha, Qatar; ^2^Division of Epidemiology and Biostatistics, European Institute of Oncology, Milan, Italy; ^3^Department of Internal Medicine, Hamad Medical Corporation, Doha, Qatar; ^4^Department of Surgery and Department of Family Medicine, New York Medical College, Valhalla, NY, USA

## Abstract

**Aims:**

Much of the diabetes burden is caused by its complications. This cross-sectional study aimed to determine the prevalence and risk factors for diabetic microvascular complications (retinopathy, nephropathy, and neuropathy) in a high-risk population.

**Methods:**

We collected information via a structured questionnaire and directly from the patient's record on 1034 adult type 2 diabetic patients who were attending outpatient clinics in Qatar.

**Results:**

The mean age of the patients was 55 ± 10 years, and the mean duration of diabetes was 12.4 ± 8.9 years. Forty-five percent had one or more microvascular complications. Shared risk factors for multiplicity and for individual complications included family history, severity and duration of diabetes, and hypertension, but some risk factors were specific for individual microvascular complications. Early age at onset of diabetes was strongly associated with multiplicity of complications (*P* = 0.0003).

**Conclusions:**

About half the diabetics in this high-risk population had one or more microvascular complications. Several well-established risk factors were associated with multiplicity and individual microvascular complications, but each separate microvascular complication was linked to a somewhat different constellation of risk factors.

## 1. Introduction

Bahrain, Kuwait, Oman, Qatar, Saudi Arabia, and the United Arab Emirates constitute the members of the Gulf Cooperation Council (GCC) and have high prevalence rates of type 2 diabetes [1]. In Qatar, the burden of diabetes is particularly high, with an estimated 4.35 years of disability adjusted life years directly attributable to this cause [2].

Elsewhere in the Middle East, diabetes is also prevalent, mirroring the current global epidemic of diabetes [3]. The high prevalence of obesity and lack of exercise, along with the possibility of region-specific genetic mutations associated with diabetes, may be the explanation, thereby further confirming the genetic peculiarity of some populations [4, 5].

Complications of diabetes contribute greatly to the increased mortality and morbidity associated with this disease. Diabetic complications are customarily divided into two main categories: macrovascular complications, including heart disease and stroke, and microvascular complications, which include retinopathy, nephropathy, and neuropathy. In the multinational A1chieve study, a large global study, based on nearly 68,000 type 2 diabetics, microvascular complications accounted for about half the total number of complications [6].

Microvascular complications, the focus of this report, are a leading cause of blindness, kidney failure, and lower limb amputation. It is unclear why some diabetic patients develop microvascular complications and other patients do not. Previous investigators have pointed out that the links between the various microvascular complications need further exploration [7, 8] and only limited data are available about their prevalence in Qatar.

The aims of this study performed in the type 2 diabetic adult population of Qatar were to (1) examine the frequency and overlap of microvascular complications, (2) determine risk factors for individual diabetic complications, and (3) determine if smoking, a risk factor for diabetes, was also associated with microvascular complications.

## 2. Materials and Methods

### 2.1. Study Subjects

The information in this report came from a cross-sectional survey conducted among patients attending the outpatient clinics of Hamad General Hospital, the largest tertiary care hospital in Doha, the capital of Qatar. Data were collected from June 2013 to January 2015 using a detailed questionnaire (supplementary file available on request) covering patient background information, health status, tobacco use, chronic disease history, and family history. Trained personnel from the Division of Global and Public Health (now Institute for Population Health), Weill Cornell Medicine-Qatar, completed the questionnaire while interviewing patients at the time of their clinic visit. The patients were recruited from the diabetic, endocrine, gastroenterology, ophthalmology, nephrology, dietetic, podiatry, and smoking cessation clinics. The inclusion criteria for recruitment were age ≥ 18 and previous diagnosis of diabetes. Exclusion criteria included subjects less than 18 years of age, subjects diagnosed with type I diabetes, and those with gestational diabetes. In addition to information obtained directly from patients, the interviewer also collected information from the patient's chart relating to current findings on physical examination, medication use, and laboratory data. Detailed smoking questions were included in the questionnaire because smoking is a known risk factor for diabetes, but its relationship to various complications has not been well documented. Because of the low prevalence of smoking in the female population of this region (less than one percent of Qatari women smoke, and the prevalence of smoking in non-Qatari women is only about 3%) [9], we prematurely stopped the enrollment of female diabetic patients in the study on May 15, 2014, concentrating our resources and effort to male diabetics. To achieve diagnostic homogeneity in the study, we limited participants to adults (age 18 or more) diagnosed with type 2 diabetes.

### 2.2. Ethical Considerations

The study was reviewed and approved by the Research Office at Weill Cornell Medicine-Qatar and the Research Committee at Hamad Medical Corporation. Patient participation in the study was entirely voluntary, and less than 2% of patients who were approached opted out of the study. Prior to the interview, a statement regarding the study embodying the elements of consent was read out to the subjects and verbal consent was obtained from each recruited subject.

### 2.3. Statistical Analysis

The study sample size (800 men) was calculated to detect a 10% difference in the prevalence of complications among male diabetic smokers, corresponding to an odds ratio = 1.5, assuming that 45% of men ever smoked and that the prevalence of complications is 50% in nonsmokers, using a two-sided *Z* test with pooled variance. Association between sociodemographic, lifestyle, and past medical history characteristics and the number of complications was assessed with the Mantel-Haenszel test for trend (categorical variables) and analysis of variance (ANOVA) for continuous variables. Odds ratios (OR) and 95% confidence intervals (CI) for the association between patients' characteristics and microvascular disease were estimated using logistic regression models adjusted for age, gender, and duration of diabetes. Multivariable logistic regression was then used to assess independence of the associations, fitting together all the variables that were associated with outcome in the previous analysis. Factors considered for inclusion in the multivariable analysis included the following: age (per 10-year increase), gender, duration of diabetes (10 or more years versus less than 10 years), education level, first degree family history of diabetes (yes versus no), insulin treatment (yes versus no), body mass index (per 5 kg/m^2^), history of hypertension under treatment, blood pressure (stage 1 or 2 hypertension (≥140/≥90) versus normal or prehypertension), LDL level (per 1 unit increase), HbA1c blood level (per 1 unit increase), and high creatinine level (>106 versus ≤106 *μ*mol/L). All analyses were performed with SAS version 9.4 (Cary, NC). All *P* values were two-sided. *P* values < 0.05 were considered statistically significant.

## 3. Results and Discussion

### 3.1. Results

Table 1 contains information about the 1034 patients with type 2 diabetes mellitus who were included in the analysis. The mean age (±standard deviation) of patients was 55 years ± 10 years (range 18–84). The average patient age at onset of diabetes was 43 ± 11 years, the average duration of diabetes was 12.4 ± 9.0 years, and the mean glycated hemoglobin (HbA1c) level was 8.3 ± 1.8%. Forty-six percent of male patients were current or former smokers, while only 2 (0.8%) of female patients ever smoked. Gender-specific participants' characteristics are available in Supplementary Tables 1a and 1b.

When compared to patients with no complications, several variables were associated with multiplicity of complications. These included age, gender, education, family history, duration, and age at onset of diabetes, HbA1c level, diabetic therapy, LDL, creatinine levels, and elevated systolic blood pressure.

**Figure 1:**
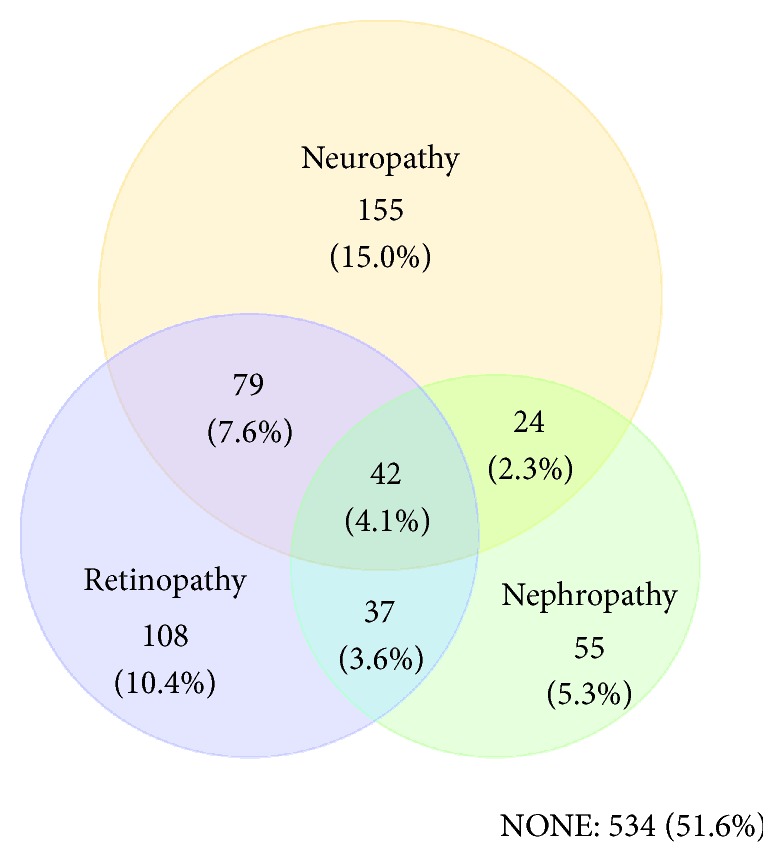
Prevalence of major microvascular complications in 1034 patients with type 2 diabetes in Qatar.

Table 2 and Figure 1 provide information about the frequency of individual and combinations of complications, overall and by gender. We stratified microvascular complications into the same major groups used by other authors: nephropathy, neuropathy, and retinopathy [4]. Microvascular complications were present at the time of the survey in 48.4% (*n* = 500) of patients; 30.8% (*n* = 318) had one complication; 13.5% (*n* = 140) had two microvascular complications, and only 4.1% (*n* = 42) had all three microvascular complications. Significantly more women than men reported any microvascular complication (61.0% versus 44.6%, *P* < 0.0001).

Three hundred (29.0%) patients reported neuropathy, 266 (25.7%) retinopathy, and 158 (15.3%) nephropathy. Female patients had higher prevalence of neuropathy (45.8% versus 24.1%, *P* < 0.0001) and retinopathy (32.2% versus 23.8%, *P* = 0.01) but lower prevalence of nephropathy (10.6% versus 16.7%, *P* = 0.02) than male patients.

The mean age at development of all three microvascular complications was similar and ranged from 53.3 ± 9.2 years (neuropathy) to 55.2 ± 10.6 years (nephropathy). Similarly, the mean interval between the diagnosis of diabetes and the development of microvascular complications was also similar, ranging from 12.9 ± 8.5 years (neuropathy) to 14.4 ± 8.1 years (retinopathy).

Table 3 presents the results of a multivariable analysis of all factors that were associated with diabetes in after sole adjustment for age, gender, and duration of diabetes (results available in Supplementary Table 2). Several variables were significantly associated with one or more microvascular complications, but HbA1c was the only variable significantly associated with all three complications in the fully adjusted model. Each of the three major microvascular complications had a distinct pattern of associated risk factors.

### 3.2. Discussion

This study focused on the prevalence of major complications in previously diagnosed adult male diabetics attending outpatient clinics at Hamad General Hospital that is a major provider of health care for the diabetic population of Doha, the capital of Qatar. In these adult type 2 diabetic patients, about half developed one or more microvascular complications 12–14 years after the onset of diabetes. In patients who did develop complications, about two-thirds developed a single complication and one-third developed two or more complications.

The role of smoking as an important risk factor for diabetes needs further emphasis: about half the subjects in this group of male diabetics were current or ex-smokers. The increased prevalence of smoking in these patients is understandable since smoking is known to be an important risk factor for diabetes [10]. However, even though smoking is known to be related with diabetes, smoking status (ever versus never) was unrelated to the presence or absence of microvascular complications nor to the development of any single microvascular complication, perhaps because smoking has equal effects on the microcirculation in the various sites were microvascular complications are diagnosed.

In general, factors that were associated with multiplicity of microvascular complications listed in Table 1 were similar to those related to each individual complication. Age at onset of diabetes was strongly associated with increasing number of complications. Multiple complications were inversely associated with age: patients with two or more complications were significantly younger than patients without any microvascular complications. In other regions, studies that have looked at age of onset of diabetes have found that early onset diabetes, usually defined as <45 years, increases disease severity. This observation suggests that patients with early onset of diabetes have a more aggressive disease and are prone to develop more complications at an earlier age [11–13]. Genetic determinants of diabetes are likely to be more common in patients with early onset of diabetes [14]. Our report agrees with other studies that the early onset of diabetes could be a helpful prognostic variable to predict the development of microvascular complications.

HbA1c was the only factor significantly associated with all three microvascular complications, although hypertension (current or under treatment) achieved borderline significance (*P* = 0.06). This finding implies that careful diabetic control is a worthwhile and achievable goal for minimizing the excess disability and costs associated with diabetes [15]. Of interest, each individual microcirculatory complication was associated with its own somewhat different pattern of risk factors. In addition to HbA1c levels and hypertension, these included patient age, family history, duration of diabetes, insulin use, and body mass index. In particular, concerning body mass index, it is well known that obesity predisposes to type 2 diabetes, a condition for which even a moderate weight loss can improve insulin resistance and chronic hyperglycemia, both of which are related to microvascular complications [16, 17].

This study has several weaknesses: it is cross-sectional and therefore not the best design for studying temporal changes. Although the possibility of interviewer bias exists, we believe that it should not affect our main findings since information about diabetic complications and treatment are not subjective and were corroborated with data retrieved from clinical charts. Also, participants were entirely voluntary and interviews were conducted by trained personnel. Since it is based on a clinic population, we have no information about the number of deaths or the causes of antecedent mortality, which could skew our results. As an example, smoking could be a major causative factor for deaths in this clinic population. Since this is a single institution study, the results and in particular the prevalence of complications may not be generalized; however, it was conducted in the largest tertiary care hospital in Doha, which is the most representative institution in the country. Furthermore, we report only microvascular and not macrovascular complications.

The study strengths are that the information comes from a carefully followed group of adult diabetics from a region with a high prevalence of diabetes. The Qatari and non-Qatari were similar, indicating that the findings are likely to be generalizable to the large population of male diabetics in the Middle East and perhaps to other regions. Another strength of the study is that it provides a road map to help guide diabetologists to compare and assess the likelihood of development of microvascular complications in their patients, as well as to determine the relative importance of individual risk factors for the individual type of complications.

In conclusion, this report provides information about the prevalence of individual and multiple microvascular complications among diabetics in Qatar, which has among the highest incidence rates of diabetes globally. The frequency of individual microvascular complications is generally similar to reports based on similarly aged patients from the same region [18–21] and from other regions [22–24]. However, unlike many previous reports which focus on a single complication, this report provides information for clinicians about the timing, the overlap, the pattern of development, and the risk factors associated with multiple microvascular complications. This information can be useful to diabetologists and clinicians responsible for the management of the large group of diabetics who develop microvascular complications.

## Figures and Tables

**Table 1:** Participant characteristics.

	All	Number of complications^∗^			
	Patients	None	1	≥2	*P* value ^∗∗^
	*N* (%)	*N* (%)	*N* (%)	*N* (%)	
All	1034 (100)	534 (100)	318 (100)	182 (100)	
Age (mean, SD), years	55 ± 10	53 ± 11	57 ± 10	59 ± 9	<0.0001
Sex					
Males	798 (77.2)	442 (82.8)	228 (71.7)	128 (70.3)	
Females	236 (22.8)	92 (17.2)	90 (28.3)	54 (29.7)	<0.0001
Nationality					
Qatari	419 (40.5)	202 (37.8)	127 (39.9)	90 (49.5)	
Non-Qatari	615 (59.5)	332 (62.2)	191 (60.1)	92 (50.5)	0.01
Education					
≤High school	538 (52.0)	250 (46.8)	170 (53.5)	118 (64.8)	
>High school	496 (48.0)	284 (53.2)	148 (46.5)	64 (35.2)	<0.0001
Household monthly income					
<$2750	257 (33.3)	140 (32.7)	74 (32.3)	43 (37.1)	
$2750–5500	235 (30.4)	126 (29.4)	72 (31.4)	37 (31.9)	
≥$5500	281 (36.3)	162 (37.9)	83 (36.2)	36 (31.0)	0.26
Family history of diabetes					
No	348 (35.1)	211 (40.5)	100 (33.8)	37 (21.3)	
Yes	643 (64.9)	310 (59.5)	196 (66.2)	137 (78.7)	<0.0001
Duration of diabetes					
Less than 10 years	453 (43.8)	330 (61.8)	102 (32.1)	21 (11.5)	
10 or more years	581 (56.2)	204 (38.2)	216 (67.9)	161 (88.5)	<0.0001
Age at onset of diabetes, years	43 ± 11	44 ± 10	43 ± 11	39 ± 11	<0.0001
HbA1c, percent	8.3 ± 1.8	8.0 ± 1.8	8.4 ± 1.7	9.0 ± 1.9	<0.0001
Treatment for diabetes					
Drug	549 (53.6)	338 (64.1)	162 (51.3)	49 (26.9)	
Insulin	436 (42.5)	161 (30.6)	145 (45.9)	130 (71.4)	
Other	40 (3.9)	28 (5.3)	9 (2.9)	3 (1.7)	<0.0001
BMI, kg/m^2^	30.8 ± 6.5	30.1 ± 6.4	31.1 ± 6.4	32.1 ± 6.9	0.001
Normal weight	163 (17.5)	94 (19.8)	45 (15.6)	24 (14.5)	
Overweight	320 (34.5)	177 (37.3)	92 (31.9)	51 (30.7)	
Obese	446 (48.0)	204 (42.9)	151 (52.4)	91 (54.8)	0.005
Smoking status					
Never smoked regularly	665 (64.3)	335 (62.7)	202 (63.5)	128 (70.3)	
Ever smoke regularly	369 (35.7)	199 (37.3)	116 (36.5)	54 (29.7)	0.10
LDL, mmol/L	2.5 ± 0.9	2.6 ± 0.9	2.4 ± 0.9	2.4 ± 1.0	0.02
Cholesterol, mmol/L	4.4 ± 1.1	4.5 ± 1.1	4.3 ± 1.1	4.2 ± 1.3	0.05
Creatinine, *μ*mol/L	93 ± 52	83 ± 34	94 ± 49	120 ± 80	<0.0001
≤106 *μ*mol/L	819 (80.5)	475 (91.4)	241 (76.5)	103 (56.6)	
>106 *μ*mol/L	198 (19.5)	45 (8.6)	74 (23.5)	79 (43.4)	<0.0001
History of hypertension					
No	361 (34.9)	236 (44.2)	89 (28.0)	36 (19.8)	
Yes	673 (65.1)	298 (55.8)	229 (72.0)	146 (80.2)	<0.0001
Systolic BP, mmHg	138 ± 18	136 ± 17	140 ± 19	143 ± 19	<0.0001
Diastolic BP, mmHg	76 ± 11	77 ± 10	77 ± 12	74 ± 10	0.01

^∗^Retinopathy, nephropathy, and neuropathy following the diagnosis of diabetes. ^∗∗^ *P* value using the Mantel-Haenszel test for trend for categorical variables and ANOVA for continuous variables. SD: standard deviation; HbA1c: hemoglobin A1c; LDL: low-density lipoprotein; BP: blood pressure.

**Table 2:** Prevalence of major microvascular complications in 1034 patients with type 2 diabetes in Qatar, by gender.

	All	Males	Females	*P* value
	*N* (%)	*N* (%)	*N* (%)	
*Total number of patients*	1034 (100)	798 (100)	236 (100)	
*Number of complications*				<0.0001
None	534 (51.6)	442 (55.4)	92 (39.0)	
One complication	**318 (30.8)**	**228 (28.6)**	**90 (38.1)**	
Retinopathy	108 (10.4)	81 (10.2)	27 (11.4)	0.55
Nephropathy	55 (5.3)	50 (6.3)	5 (2.1)	0.01
Neuropathy	155 (15.0)	97 (12.2)	58 (24.6)	<0.0001
Two complications	**140 (13.5)**	**97 (12.2)**	**43 (18.2)**	
Retinopathy + nephropathy	37 (3.6)	33 (4.1)	4 (1.7)	0.11
Retinopathy + neuropathy	79 (7.6)	45 (5.6)	34 (14.4)	<0.0001
Nephropathy + neuropathy	24 (2.3)	19 (2.4)	5 (2.1)	1.00
Three complications	**42 (4.1)**	**31 (3.9)**	**11 (4.7)**	0.58
*Patterns of combined complications*				
Retinopathy (±other complications)	266 (25.7)	190 (23.8)	76 (32.2)	0.01
Nephropathy (±other complications)	158 (15.3)	133 (16.7)	25 (10.6)	0.02
Neuropathy (±other complications)	300 (29.0)	192 (24.1)	108 (45.8)	<0.0001

**Table 3:** Multivariable analysis of factors associated with microvascular complications.

Variable	Retinopathy		Nephropathy		Neuropathy^∗^	
	OR (95% CI)	*P* value	OR (95% CI)	*P* value	OR (95% CI)	*P* value
Age (per 10 years)	**1.19 (1.00–1.43)**	**0.05**	0.87 (0.69–1.11)	0.27	1.18 (1.00–1.39)	0.06
Gender (female versus male)	1.34 (0.92–1.95)	0.13	0.95 (0.53–1.70)	0.85	**2.07 (1.44–2.97)**	**<0.0001**
Duration of DM ≥ 10 years	**3.83 (2.56–5.72)**	**<0.0001**	1.48 (0.87–2.49)	0.15	**2.77 (1.92–3.99)**	**<0.0001**
Education (>high school)	0.79 (0.57–1.10)	0.16	**0.61 (0.38–0.96)**	**0.03**	—	
Family history of DM (yes versus no)	—		—		**1.63 (1.15–2.30)**	**0.006**
HbA1c level (per % increase)	**1.14 (1.04–1.25)**	**0.006**	**1.15 (1.00–1.33)**	**0.05**	**1.12 (1.02–1.23)**	**0.01**
DM treatment (insulin versus drug)	**1.61 (1.14–2.26)**	**0.006**	**2.05 (1.25–3.39)**	**0.005**	1.26 (0.90–1.75)	0.18
Body mass index (per 5 kg/m^2^)	—		—		**1.29 (1.14–1.47)**	**<0.0001**
LDL (per mmol/L increase)	—		0.82 (0.64–1.05)	0.11	—	
Creatinine (>106 versus ≤106 *μ*mol/L)	**1.63 (1.11–2.41)**	**0.01**	**23.9 (14.7–38.8)**	**<0.0001**	1.36 (0.93–1.99)	0.12
Hypertension or high BP	**1.87 (1.29–2.71)** ^**1**^	**0.001**	**2.03 (1.15–3.59)** ^**1**^	**0.01**	1.31 (0.97–1.78)^2^	0.08

Odds ratios (OR) and 95% confidence intervals (CI) obtained from multivariable logistic regression model with all displayed variables fitted simultaneously. ^∗^Foot ulcer and amputation. ^1^History of hypertension, undergoing treatment. ^2^Systolic blood pressure ≥ 140 or diastolic blood pressure ≥ 90 at time of survey

## Supplementary Materials

Supplementary Materials Supplementary data include characteristics of male and female participants and analysis of factors associated with microvascular complications, adjusted for age, sex, and duration of diabetes.
