# Supplementary material for: Risk Factors for Microvascular Complications of Diabetes in a High-Risk Middle East Population
Source: J Diabetes Res. 2018 Jul 2;2018:8964027. doi: 10.1155/2018/8964027 (PMC6051261; doi:10.1155/2018/8964027)
Supplement: Supplementary Materials — Supplementary data include characteristics of male and female participants and analysis of factors associated with microvascular complications, adjusted for age, sex, and duration of diabetes. [file 8964027.f1.docx]

**Supplementary Table 1a.** Characteristics of male participants

| Age (mean, SD), years | 55±11 | 53±11 | 58±10 | 59±9 | <.0001 |
| --- | --- | --- | --- | --- | --- |
|  |  |  |  |  |  |
| Nationality |  |  |  |  |  |
| Qatari | 251 (31.5) | 140 (31.7) | 68 (29.8) | 43 (33.6) |  |
| Non-Qatari | 547 (68.5) | 302 (68.3) | 160 (70.2) | 85 (66.4) | 0.75 |
| Education |  |  |  |  |  |
| ≤High school | 368 (46.1) | 193 (43.7) | 103 (45.2) | 72 (56.3) |  |
| >High school | 430 (53.9) | 249 (56.3) | 125 (54.8) | 56 (43.7) | 0.02 |
| Household monthly income |  |  |  |  |  |
| <2,750$ | 234 (29.3) | 130 (34.5) | 63 (33.0) | 41 (41.8) |  |
| 2,750-5500$ | 210 (26.3) | 112 (29.7) | 67 (35.1) | 31 (31.6) |  |
| ≥5,500$ | 222 (27.8) | 135 (35.8) | 61 (31.9) | 26 (26.5) | 0.11 |
| Family history of diabetes |  |  |  |  |  |
| No | 303 (39.4) | 193 (44.6) | 81 (38.0) | 29 (23.6) |  |
| Yes | 466 (60.6) | 240 (55.4) | 132 (62.0) | 94 (76.4) | <0.0001 |
| Duration of diabetes |  |  |  |  |  |
| Less than 10 years | 365 (45.7) | 274 (62.0) | 74 (32.5) | 17 (13.3) |  |
| 10 or more years | 433 (54.3) | 168 (38.0) | 154 (67.5) | 111 (86.7) | <0.0001 |
| Age at onset of diabetes, years | 43±11 | 44±10 | 44±11 | 40±11 | 0.0003 |
| HbA1c, percent | 8.2±1.8 | 7.9±1.7 | 8.3±1.6 | 9.0±1.9 | <0.0001 |
| Treatment for diabetes |  |  |  |  |  |
| Drug | 446 (55.9) | 285 (65.5) | 122 (53.7) | 39 (30.5) |  |
| Insulin | 310 (38.8) | 125 (28.7) | 99 (43.6) | 86 (67.2) |  |
| Other | 34 ( 4.3) | 25 ( 5.8) | 6 ( 2.6) | 3 ( 2.3) | <0.0001 |
| BMI, Kg/m^2^ | 29.5±6.0 | 29.2±6.0 | 29.5±5.8 | 30.5±6.4 | 0.11 |
| Normal weight | 152 (21.4) | 87 (22.4) | 43 (21.1) | 22 (18.6) |  |
| Overweight | 176 (38.9) | 156 (40.2) | 77 (37.7) | 43 (36.4) |  |
| Obese | 292 (39.7) | 145 (37.4) | 84 (41.2) | 53 (44.9) | 0.16 |
| Smoking status |  |  |  |  |  |
| Never smoked regularly | 431 (54.0) | 244 (55.2) | 113 (49.6) | 74 (57.8) |  |
| Ever smoke regularly | 367 (46.0) | 198 (44.8) | 115 (50.4) | 54 (42.2) | 0.97 |
| LDL, mmol/L | 2.5±1.0 | 2.5±0.9 | 2.3±0.9 | 2.4±1.0 | 0.03 |
| Cholesterol, mmol/L | 4.3±1.2 | 4.4±1.1 | 4.2±1.1 | 4.2±1.4 | 0.07 |
| Creatinine, µmol/L | 100±50 | 88±35 | 105±50 | 133±72 | <0.0001 |
| ≤106 µmol/L | 598 (76.6) | 384 (89.7) | 157 (69.8) | 57 (44.5) |  |
| >106 µmol/L | 183 (23.4) | 44 (10.3) | 68 (30.2) | 71 (55.5) | <0.0001 |
| History of hypertension |  |  |  |  |  |
| No | 282 (35.3) | 197 (44.6) | 63 (27.6) | 22 (17.2) |  |
| Yes | 516 (64.7) | 245 (55.4) | 165 (72.4) | 106 (82.8) | <0.0001 |
| Systolic BP, mmHg | 139±18 | 136±17 | 141±19 | 145±19 | <0.0001 |
| Diastolic BP, mmHg | 77±10 | 77±10 | 78±11 | 76±10 | 0.37 |

* Includes retinopathy, nephropathy and neuropathy following the diagnosis of diabetes.

** P-value using the Mantel-Haenszel test for trend for categorical variables, ANOVA for continuous variables
Abbreviations: SD, standard deviation; HbA1c, Hemoglobin A1c; LDL, Low Density Lipoprotein; BP, Blood pressure

**Supplementary Table 1b.** Characteristics of female participants

|  | All | Number of complications* | | | | |
| --- | --- | --- | --- | --- | --- | --- |
|  | **Patients** | | **None** | **1** | **>2** | **P-value**** |
|  | **N (%)** | | **N (%)** | **N (%)** | **N (%)** |  |
| ALL | 236 (100) | | 92 (100) | 90 (100) | 54 (100) |  |
| Age (mean, SD), years | 55±10 | | 52±11 | 56±10 | 59±8 | <0.0001 |
|  |  | |  |  |  |  |
| Nationality |  | |  |  |  |  |
| Qatari | 168 (71.2) | | 62 (67.4) | 59 (65.6) | 47 (87.0) |  |
| Non-Qatari | 68 (28.8) | | 30 (32.6) | 31 (34.4) | 7 (13.0) | 0.02 |
| Education |  | |  |  |  |  |
| ≤High school | 170 (72.0) | | 57 (62.0) | 67 (74.4) | 46 (85.2) |  |
| >High school | 66 (28.0) | | 35 (38.0) | 23 (25.6) | 8 (14.8) | 0.002 |
| Household monthly income |  | |  |  |  |  |
| <2,750$ | 23 (21.5) | | 10 (19.6) | 11 (28.9) | 2 (11.1) |  |
| 2,750-5500$ | 25 (23.4) | | 14 (27.5) | 5 (13.2) | 6 (33.3) |  |
| ≥5,500$ | 59 (55.1) | | 27 (52.9) | 22 (57.9) | 10 (55.6) | 0.74 |
| Family history of diabetes |  | |  |  |  |  |
| No | 45 (20.3) | | 18 (20.5) | 19 (22.9) | 8 (15.7) |  |
| Yes | 177 (79.7) | | 70 (79.5) | 64 (77.1) | 43 (84.3) | 0.59 |
| Duration of diabetes |  | |  |  |  |  |
| Less than 10 years | 88 (37.3) | | 56 (60.9) | 28 (31.1) | 4 (7.4) |  |
| 10 or more years | 148 (62.7) | | 36 (39.1) | 62 (68.9) | 50 (92.6) | <0.0001 |
| Age at onset of diabetes, years | 41±10 | | 43±10 | 42±10 | 38±9 | 0.01 |
| HbA1c, percent | 8.6±1.9 | | 8.3±1.9 | 8.7±1.9 | 9.0±1.7 | 0.09 |
| Treatment for diabetes |  | |  |  |  |  |
| Drug | 103 (43.8) | | 53 (57.6) | 40 (44.9) | 10 (18.5) |  |
| Insulin | 126 (53.6) | | 36 (39.1) | 46 (51.7) | 44 (81.5) |  |
| Other | 6 (2.6) | | 3 (3.3) | 3 (3.4) | 0 (0.0) | <0.0001 |
| BMI, Kg/m^2^ | 34.9±6.5 | | 34.0±6.6 | 35.1±6.3 | 36.1±6.7 | 0.20 |
| Normal weight | 11 (5.0) | | 7 (8.1) | 2 (2.4) | 2 (4.2) |  |
| Overweight | 44 (20.1) | | 21 (24.1) | 15 (17.9) | 8 (16.7) |  |
| Obese | 164 (74.9) | | 59 (67.8) | 67 (79.8) | 38 (79.2) | 0.08 |
| Smoking status |  | |  |  |  |  |
| Never smoked regularly | 234 (99.2) | | 91 (98.9) | 89 (98.9) | 54 (100) |  |
| Ever smoke regularly | 2 (0.8) | | 1 (1.1) | 1 (1.1) | 0 (0.0) | 0.53 |
| LDL, mmol/L | 2.6±0.8 | | 2.7±0.8 | 2.6±0.8 | 2.4±0.8 | 0.04 |
| Cholesterol, mmol/L | 4.6±1.0 | | 4.8±1.0 | 4.6±1.0 | 4.3±1.0 | 0.03 |
| Creatinine, µmol/L | 70±51 | | 60±18 | 67±32 | 90±92 | 0.002 |
| ≤106 µmol/L | 221 (93.6) | | 91 (98.9) | 84 (93.3) | 46 (82.2) |  |
| >106 µmol/L | 15 (6.4) | | 1 (1.1) | 6 (6.7) | 8 (14.8) | 0.001 |
| History of hypertension |  | |  |  |  |  |
| No | 79 (33.5) | | 39 (42.4) | 26 (28.9) | 14 (25.9) |  |
| Yes | 157 (66.5) | | 53 (57.6) | 64 (71.1) | 40 (74.1) | 0.03 |
| Systolic BP, mmHg | 138±18 | | 136±15 | 139±20 | 138±17 | 0.50 |
| Diastolic BP, mmHg | 74±11 | | 75±9 | 75±13 | 70±9 | 0.02 |

* Includes retinopathy, nephropathy and neuropathy following the diagnosis of diabetes.

** P-value using the Mantel-Haenszel test for trend for categorical variables, ANOVA for continuous variables
Abbreviations: SD, standard deviation; HbA1c, Hemoglobin A1c; LDL, Low Density Lipoprotein; BP, Blood pressure

**Supplementary Table 2.** Analysis of factors associated with microvascular complications, adjusted for age, sex and duration of diabetes.

| **Variable** | **Strata** | **Retinopathy** | | **Nephropathy** | | **Neuropathy*** | |
| --- | --- | --- | --- | --- | --- | --- | --- |
|  |  | **OR (95% CI)** | **P-value** | **OR (95% CI)** | **P-value** | **OR (95% CI)** | **P-value** |
| Age | per 10 years | **1.26 (1.07-1.48)** | **0.005** | **1.35 (1.12-1.62)** | **0.002** | **1.15 (0.99-1.34)** | **0.07** |
| Gender | F vs M | **1.43 (1.02-2.02)** | **0.04** | **0.55 (0.35-0.87)** | **0.01** | **2.81 (2.03-3.88)** | **<0.0001** |
| Duration of DM | ≥10 vs. <10 years | **5.38 (3.69-7.84)** | **<0.0001** | **2.22 (1.48-3.32)** | **0.0001** | **3.59 (2.57-5.01)** | **<0.0001** |
| Nationality | Non-Qatari vs. Qatari | 0.86 (0.62-1.19) | 0.36 | 1.27 (0.87-1.85) | 0.22 | 0.93 (0.68-1.27) | 0.66 |
| Education | >high school vs. ≤high school | **0.72 (0.53-0.99)** | **0.04** | **0.61 (0.42-0.87)** | **0.007** | 0.92 (0.689-1.25) | 0.60 |
| Family history of DM | Yes vs. no | 1.21 (0.86-1.70) | 0.28 | 1.02 (0.69-1.49) | 0.93 | **1.70 (1.22-2.37)** | **0.002** |
| HbA1c level | Per % | **1.20 (1.10-1.31)** | **<0.0001** | **1.16 (1.05-1.28)** | **0.003** | **1.15 (1.06-1.25)** | **0.0009** |
| Diabetes treatment | Insulin vs. drug | **1.96 (1.42-2.71)** | **<0.0001** | **2.30 (1.56-3.38)** | **<0.0001** | **1.59 (1.17-2.16)** | **0.003** |
|  | Other vs. drug | 0.91 (0.30-2.74) | 0.86 | **2.48 (1.02-6.05)** | **0.05** | 0.69 (0.26-1.84) | 0.46 |
| Body Mass Index | per 5 kg/m^2^ | 1.07 (0.94-1.21) | 0.32 | 1.09 (0.94-1.27) | 0.24 | **1.31 (1.16-1.48)** | **<0.0001** |
| Smoking | Ever vs. never | 0.79 (0.55-1.12) | 0.18 | 0.88 (0.60-1.29) | 0.50 | 1.10 (0.79-1.55) | 0.57 |
| Cholesterol | per 1 mmol/L increase | 0.92 (0.80-1.07) | 0.28 | 0.87 (0.73-1.04) | 0.12 | 1.11 (0.97-1.27) | 0.13 |
| LDL | per 1 mmol/L increase | 0.92 (0.77-1.10) | 0.35 | **0.79 (0.64-0.98)** | **0.03** | 1.14 (0.97-1.34) | 0.12 |
| Creatinine | >106 vs. ≤106 µmol/L | **1.94 (1.34-2.82)** | **0.0005** | **25.8 (16.2-41.1)** | **<0.0001** | **1.46 (1.01-2.11)** | **0.04** |
| Hypertension history^1^ | Yes vs. No | **2.11 (1.47-3.02)** | **<0.0001** | **3.10 (1.92-4.99)** | **<0.0001** | 0.99 (0.72-1.37) | 0.92 |
| High blood pressure^2^ | High vs. Normal | 1.53 (1.13-2.08) | 0.006 | **1.53 (1.07-2.189)** | **0.02** | **1.47 (1.10-1.97)** | **0.009** |

*Includes foot ulcer and amputation.

^1^History of hypertension, undergoing treatment. ^2^Systolic blood pressure ≥140 or diastolic blood pressure ≥90 at time of survey

Abbreviations: DM, diabetes mellitus; HbA1c, Hemoglobin A1c; LDL, Low Density Lipoprotein.
